# Garlic essential oil supplementation modulates colonic microbiota compositions and regulates immune response in weaned piglets

**DOI:** 10.1016/j.heliyon.2023.e18729

**Published:** 2023-07-27

**Authors:** Bei Cheng, Mingyong Huang, Tiantian Zhou, Qingqing Deng, Teketay Wassie, Tao Wu, Xin Wu

**Affiliations:** a Hubei Key Laboratory of Animal Nutrition and Feed Science, Wuhan Polytechnic University, Wuhan 430023, China; b CAS Key Laboratory of Agro-Ecological Processes in Subtropical Region, Institute of Subtropical Agriculture, Chinese Academy of Sciences, Changsha 410125, China; c Hunan Tianxiang Biotechnology Co., Ltd, Shaoyang 422000, China; d Henan Institute of Science and Technology, College of Animal Science and Veterinary Medicine, Xinxiang 453004, China; e Oregon Health and Science University, School of Medicine, department of Molecular Microbiology and Immunology, Portland, OR, USA

**Keywords:** Garlic essential oil, Microbiota, Immune response, Weaned piglets, Virulence factor

## Abstract

The objective of this study was to investigate the colonic microbiome compositions and immune response and reveal their correlations in weaned piglets fed with garlic essential oil (GEO). Twelve 21-day-old crossbred piglets with the same parity and similar weight (BW = 7.07 ± 0.37 Kg) were randomly divided into control and experimental groups based on BW and sex, which fed either a basal diet (CON group), or a basal diet supplemented with 1.5 g/kg GEO (GEO group). UHPLC-QE-MS showed the main component of GEO were belonged to carbohydrates, organic acid, flavonoids, phenylpropanoids and terpenoids. GEO decreased serum IL-1β, IL-8 content and the down-regulated mRNA expression of *IFN-γ*, *TLR2* in jejunal mucosa but increased serum IgG, IL-4 content and up-regulated the mRNA expression of *IL-4*, *IL-1β*, *TNF-α* in ileal mucosa. What's more, the metagenomic analysis demonstrated that GEO increased the abundance of *Bacteroidetes*, *Euryarchaeota* and *Spirochaetes*, while decreased the abundance of *Firmicutes* and *Actinobacteria* at Phylum level and *Selenomonas_boris*, *Selenomonadaceae_bacterium_DSM_108025*, *Clostridiales_bacterium* and *Phascolarctobacterium_succinatutens* at species level. Notably, the main function pathway of virulence factor (VFDB) enriched in GEO group were Fibronection-binding protein, Zn^++^ metallophrotease and Capsular polysaccharide, while the main function pathway of VFDB enriched in CON group were heme biosynthesis, Lap and FeoAB. Spearman correlation analysis indicated the *Spirochaetes* had a positive association with IL-6 and IL-4. *Acinobacteria* was positively correlated with IL-1β, while negative with the IL-6; In addition, *Euryarchaeota* had a positive correlation with IL-4, but a negative correlation with IL-1β; *Tenericutes* was negative with IL-8; *Phascolarcolarctobacterium_succinatutens* and was negative with IL-6; *Ruminococcaceae_bacterium* was negative with TNF-α. While *Selenomonadaceae_bacterium_DSM_*108025 had a positive correlation with IL-8. In conclusion, our results uncovered that immune regulation effects of GEO may be associated with the microbiome compositions in response to GEO.

## 1 Introduction

Weaning stress always accompanied with diarrhea in weaned piglets due to inflammation and intestinal flora disorders, which may result in changes in mRNA levels of small intestinal cytokines and tight junction proteins [[1], [2], [3], [4], [5], [6]]. Over the past few decades, antibiotics or zinc oxide (ZnO) were widely added into the feed of weaned piglets to relieve diarrhea after weaning, however, the usage of antibiotics and ZnO leads to environmental pollution and development of antibiotic resistance bacteria, which seriously threatens human public health [7,8]. And they might seriously damage the function of pig immune cells, inhibits cellular immunity and humoral immunity, and reduces immunity and disease resistance of piglets. Therefore, searching for a green and healthy alternative is critical.

Essential oils are plant extracts used for various pharmacological purposes. Due to their nature, non-toxic side effects and no drug resistance, they have been widely used in the pharmaceutical and food industry, and can be used as a potential substitute for antibiotics and growth promoters. Plant essential oil has antibacterial effect and has been widely used in the treatment of livestock and poultry diseases. Among these plants, garlic (*Allium sativum*) has been used as growth promoter in livestock for about 50 years. Additional, garlic has antibacterial, antiviral, antifungal, antioxidant and immunomodulatory functions [[9], [10], [11], [12]]. Dietary garlic product such as allicin are known to improve health and homeostasis by enhancing the intestinal immune function in rabbit [13] and inhibiting the pathogenic microorganism in rat [14]. Li et al. [15] found that allicin reduced serum TNF-α and IL-1β levels, colon IL-1β levels and increased serum IL-4 levels, and played an anti-inflammatory role by reducing NF-kB and inhibiting P38 and JNK pathways. The mechanism may be that allicin acts on the upstream of TLR2/4 and MyD88 then regulates downstream targets P38 and NF-kB, finally, reduced serum TNF-α and IL-1β levels, colon IL-1β levels and increased serum IL-4 levels. Revealing the effects of garlic essential oil (GEO) on a given microbial population and associations of this microbiota and its metabolites with the immune of the host plays crucial roles to develop garlic-based functional feed addictive to prevent and treat gut microbiota-related diseases. Thus, here we studied the effects of dietary GEO supplementation on the colonic microbiome compositions and immune response and reveal their correlations in weaned piglets.

## 2 Material and methods

### 2.1 Ethical statement

The animal experiments were approved by the Animal Care and the Animal Welfare Committees of the Institute of Subtropical Agriculture at the Chinese Academy of Sciences, with protocol 2015-8A.

### 2.2 Source of GEO

The GEO was produced from the natural garlic and provided by Hunan Tianxiang Biotechnology Co., Ltd (Changsha, China). The main production process of GEO was: firstly, fresh garlic (Rocambole) was mashed then soaked in water and oil to extract GEO, then fermented to deodorize, then separated, concentrated and purified. Finally, purified garlic essential oil is attached to porous multigrain starch balls made by enzyme.

### 2.3 UHPLC-QE-MS analysis of the constituents from GEO

Ultra Highly Performance Liquid Chromatography Q Exactive-mass spectrometer (GC-TOF-MS) was performed and analyzed at SHANGHAI BIOTREE BIOTECH CO., LTD.

#### 2.3.1 Sample extraction

The 100 mg sample was weighed and 500 μL extract was added (methanol: water = 4:1, internal standard concentration was 10 μg/mL). Then the samples were swirled for 30 s, homogenized at 45 Hz for 4 min, and ultra sounded in ice water bath for 1 h. After standing at −40 °C for 1 h, the samples were centrifuged at 12000 rpm/min (centrifugal force 13800 ( × g), radius 8.6 cm) at 4 °C for 15 min. Subsequently, the supernatant was passed through a 0.22 μm filter membrane and put into a fresh 2 ml tube for UHPLC–MS/MS analysis.

#### 2.3.2 LC-MS/MS conditions

LC-MS/MS analysis was performed on an UHPLC system (Vanquish, Thermo Fisher Scientific) with a Waters UPLC BEH C18 column (1.7 μm 2.1 *100 mm). The flow rate was set at 0.4 mL/min and the sample injection volume was set at 5 μL. The mobile phase consisted of 0.1% formic acid in water (A) and 0.1% formic acid in acetonitrile (B). The multi-step linear elution gradient program was as follows: 0–3.5 min, 95 - 85% A; 3.5–6 min, 85 - 70% A; 6–6.5 min, 70 - 70% A; 6.5–12 min, 70 - 30% A; 12–12.5 min, 30 - 30% A; 12.5–18 min, 30 - 0% A; 18–25 min, 0 - 0% A; 25–26 min, 0–95% A; 26–30 min, 95 - 95% A. An Orbitrap Exploris 120 mass spectrometer coupled with an Xcalibur software was employed to obtain the MS and MS/MS data based on the IDA acquisition mode. During each acquisition cycle, the mass range was from 100 to 1500, and the top four of every cycle were screened and the corresponding MS/MS data were further acquired. Sheath gas flow rate: 30 Arb, Aux gas flow rate: 10 Arb, Ion Transfer Tube Temp: 350 °C, Vaporizer Temp: 350 °C, Full ms resolution: 60000, MS/MS resolution: 15000, Collision energy: 16/38/42 in NCE mode, Spray Voltage: 5.5 kV (positive) or −4 kV (negative).

#### 2.3.3 Multivariate data processing

Raw data analysis, including peak extraction, baseline adjustment, deconvolution, alignment and integration, was performed with Chroma TOF (V 4.3x, LECO) software and LECO-FiehnRtx5 database was used for metabolite identification by matching the mass spectrum and retention index.

### 2.4 Design and Husbandry

Twelve 21-day-old healthy crossbred piglets (Duroc × Landrace × Yorkshire) with the same parity and similar weight were bought from Jiahe Agricultural Stockbreeding Co., Ltd, and randomly divided into control and experimental groups (6 weaned piglets per treatment, BW = 7.07 ± 0.37 Kg) after 5 days of per-experiment based on BW and sex, which fed either a basal diet (CON group), or a basal diet supplemented with 1.5 g/kg GEO (GEO group), and each pig was raised in a single pen. The per-experimental feeding was gradually replaced by the original farm feed with the experimental diet during the pre-experiment period. The experiment period lasted for 21 days. All the piglets have free access to feed and water. The basal diet was formulated based on the National Research Council (2012) recommendation, whose composition and nutritional levels are listed in Table 1.

**Table 1:** Composition and nutrient levels of the basal diet (DM basis) %.

Item ingredients	Content	Nutrient levels	
Corn	58.42	DE/(MJ/kg)	14.30
Soybean meal	25.00	CP (%)	19.00
Fish meal	5.00	Ca (%)	0.58
Whey powder	4.00	AP (%)	0.42
Cream powder	5.00	Lys (%)	1.20
Limestone	0.30	Met (%)	0.40
CaHPO4	1.10	Thr (%)	0.85
Moldproofant	0.10		
Antioxidant	0.02		
Vitamin premix^a^	0.04		
Choline chloride	0.08		
Mineral premix^b^	0.30		
NaCl	0.30		
Flavor	0.06		
*L*-Lys HCl	0.23		
Met	0.05		
Total	100.00		

a Provided additional vitamins per kilogram diet: VA 11 000 IU, VD3 1100 IU, VE 16 IU, VK 1 mg, pantothenate 6 mg, retinoic acid 2 mg, folic acid 0.8 mg, nicotinic acid 10 mg, thiamine 0.6 mg, VB1 0.6 mg, biotin 0.08 mg, VB12 0.03 mg.

b Provided with additional trace elements per kilogram diet: Zn 165 mg, Fe 165 mg, Mn 33 mg, Cu 16.5 mg, I 297 μg, Se 297 μg.

### 2.5 Sample collection

All piglets were anesthetized with pentobarbital sodium (50 mg/kg body weight) and bled by exsanguination. Blood samples were collected from the jugular vein in 10 mL tubes and sera were collected by centrifuging at 3500 rpm/min for 10 min. Jejunum mucosa, ileum mucosa samples and colonic content were collected and immediately stored in −80 °C for the subsequent analysis.

### 2.6 Serum cytokine and IgG analysis

Serum concentrations of interleukin-1β (IL-1β), IL-4, IL-6, IL-8, tumor necrosis alpha (TNF-α) and interferon-gamma (IFN-γ) were measured using commercial pig-specific ELISA kits (Shanghai Kexin Biotech Co., Ltd, Shanghai, China), following the kit instruction. And the serum IgG level was measured using biochemical automatic analyzer (Biochemical Analytical Instrument, Beckman CX4, Beckman Coulter Inc., Brea, CA).

### 2.7 Real-time PCR

Jejunum and ileum mucosa were homogenized under liquid nitrogen, and RNA was extracted according to Xie [16]. The mRNA expression of target genes and internal control gene (β-actin) were detected using qPCR (SYBR Green Pro Taq HS premix Q PCR kit) following the kit instruction. Briefly, 2 μL cDNA template was added to a 10 μL assay solution containing 5 μL SYBR Green mix, 2.4 μL deionized water, and 0.3 μL each for forward and reverse primers. The PCR cycle consists of an initial step of 30 s at 95 °C, followed by 40 cycles of 5 s at 95 °C and 20 s at 60 °C. The primers used in the present study are shown in Table 2, and the 2^−ΔΔCt^ method was used to calculate the relative mRNA expression levels.

**Table 2:** Primers used for quantitative polymerase chain reaction.

Target gene	Accession NO.	Nucleotide sequence of primer (5′-3′)	Size (bp)
β-actin	XM_021086047.1	F: TCTGGCACCACACCTTCT	114
		R: TGATCTGGGTCATCTTCTCAC	
TNF-α	NM_214022.1	F: TCTGCCTACTGCACTTCGAG	140
		R: GTTGATGCTCAAGGGGCCA	
IL-8	NM_213867.1	F: GACTTCCAAACTGGCTGTTGC	122
		R: ATTTGGGGTGGAAAGGTGTG	
IL-4	NM_214340.1	F: CCCGAGTGTCAAGTGGCTTA	123
		R: TGATGATGCCGAAATAGCAG	
IL-1β	NM_001302388.2	F: CCAAAGAGGGACATGGAGAA	381
		R: GGGCTTTTGTTCTGCTTGAG	
IFN-γ	NM_213948.1	F: CAGCTTTGCGTGACTTTGTG	188
		R: GATGAGTTCACTGATGGCTTT	
NF-κB	NM_001114281.1	F: GACCTGGTTTCGCTCTTG	2228
		R: TGCTGTATCCGGGTACTT	
P38 MAPK	XM_021079285.1	F: ATTCTCCGACGGTCTCAAGT	240
		R: GCCACATAGCCTGTCATT	
TLR2	XM_005653577.3	F: CTGCTCCTGTGACTTCCTGTC	81
		R: AGGTAGTTCTCCGGCCAGTC	
TLR4	NM_001293317.1	F: CCATGGCCTTTCTCTCCTG	90
		R: TCAGCTCCATGCATTGGTAA	
MyD88	NM_001099923.1	F: GCAGCTGGAACAGACCAACT	66
		R: GTGCCAGGCAGGACATCT	
GPR41	NM_001315601.1	F: TCTTCACCACCGTCTATCTCAC	398
		R: CACAAGTCCTGCCACCCTC	
GPR43	XM_021093196.1	F: CTGCCTGGGATCGTCTGTG	249
		R: CATACCCTCGGCCTTCTGG	

IL-8, Interleukin 8; IL-1β, Interleukin 1 beta; IL-4, Interleukin 4; TNF-α, Tumor necrosis factor-alpha; IFN-γ, Interferon-gamma; TLR, Toll-like receptor; MyD88, myeloid differentiation 88; NF-κB, Nuclear factor-kappa B. MAPK, mitogen activated protein kinase; GPR, G-protein coupled receptor.

### 2.8 Metagenomic analysis

#### 2.8.1 Microbial DNA extraction

To assess the colonic microbial composition in response to GEO supplementation, the colonic contents of 4 experimental weaned piglets from each group were collected and subjected to metagenomic sequencing. Microbial DNA was extracted using E.Z.N.A.® Soil DNA Kit (Omega Bio-tek, Norcross, GA, U.S.), according to the instruction of the manufacturer. The DNA concentration was determined using a NanoDrop 2000 spectrophotometer (Thermo Fisher Scientific, MA, USA), and the DNA quality was assessed by running 1% agarose gel electrophoresis. The metagenomic DNA samples were sequenced 400 bp opposite using the NovaSeq kit/HiSeq X kit on Illumina's NovaSeq platform at Mayobbio Biopharmaceutical Technologies Co., LTD. (Shanghai, China). Data analysis was performed using the Majorbio CloudPlatform online platform (www.majorbio.com). The low-quality reads were removed by fastp (https://github.com/OpenGene/fastp , version 0.20.0) [17]. Reads were compared with *Sus scrofa* genome by BWA method (https://www.ncbi.nlm.nih.gov/genome/?term=Sus) [18]. Contigs with length ≥100 bp were selected for further gene prediction and annotation. Prediction of open reading Frames (orf) of each combination contig using Meta Gene (http://metagene.cb.k.u-tokyo.ac.jp/) [19]. A non-redundant gene catalog was established by CD-HIT (http://www.bioinformatics.org/cd-hit/ , version 4.6.1) [20] with 90% sequence identity and 90% coverage. After quality control, the sequencing results were mapped to 95% identical nonredundant gene catalogs using a SOAP comparator (http://soap.genomics.org.cn/, version 2.21) [21], the gene abundance of each sample was calculated.

#### 2.8.2 Functional annotation

Representative non-redundant gene catalogs were compared with the NCBI NR database for classification annotation and Cluster of orthologous groups of proteins (COG). Functional annotations of representative genes in the virulence factor database (VFDB) (http://www.mgc.ac.cn/VFs/) were further analyzed using Diamond [22].

### 2.9 Short-chain fatty acids analysis

The short-chain volatile fatty acids (acetate, butyrate, propionate, iso-butyrate, valerate, and isovalerate) were measured from colonic content samples using the Agilent 6890 gas chromatography (Agilent Technologies, Inc, Palo Alto, CA) according to the previous study [23]. To ensure the uniformity of colonic chyme samples, freeze-dried samples were prepared by Vacuum freeze-drying machine (Hrist ALPHA 2–4/LSC, Germany) at −80 °C. Simply, the freeze-dried sample (about 1 g) was weighed into a 10 mL centrifuge tube, mixed evenly with 8 mL ddH2O, centrifuge 7000 *g* in a sealed tube at 4 °C for 10 min. The mixture of supernatant and 25% metaphosphoric acid solution (0.9 mL and 0.1 mL, respectively) was centrifuged at 20 000×*g* and 4 °C for 10 min after standing for more than 2 h in a 2 mL sealed tube at 4 °C. The supernatant was filtered by 0.45 μm polysulfone filter and analyzed by gas chromatography with Agilent 6890 (Agilent Technologies, Inc, Palo Alto, CA, USA) with a flame ionization detector and a 1.82 m × 0.2 mm I.D. glass column that was packed with 10% SP-1200/1% H3PO4 on the 80/100 Chromosorb W AW (HP, Inc., Boise, ID, USA).

### 2.10 Statistical analysis

The data was analyzed using statistical software SPSS 24.0 (SPSS IBM, New York, USA) and means were compared by student t-test. The results were expressed as means ± standard error of the means (SEM). Differences between means were considered *P* < 0.10 as a significant trend, *P* < 0.05 as significant difference, and *P* < 0.01 as extremely significant.

## 3 Results

### 3.1 Main chemical components of GEO

**Fig. 1:**
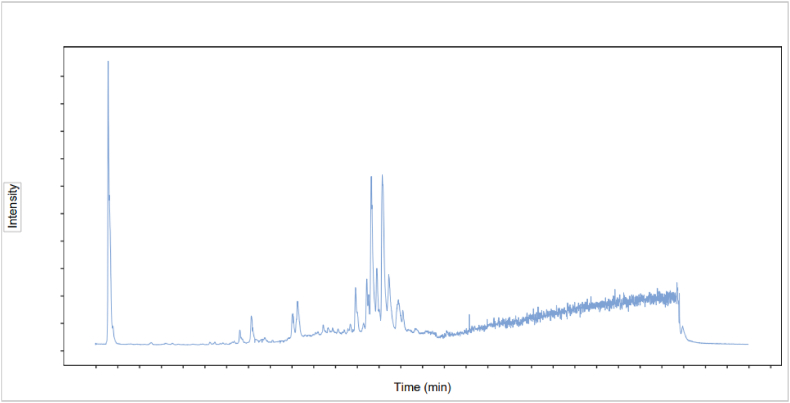
UHPLC-QE-MS total ion chromatograms of GEO.

In total, 278 certain compounds were detected in the GEO (Fig. 1), there were 18 major classes that were identified with average percent chemical composition of >1% and were displayed in Table 3. Cellobiose consisted the highest composition of the identified chemical classes at 33.12%. Other major chemical classes identified in this study included D-(+)-Malic acid, Mannose, Fumaric acid, Citric acid, Glycitein, d-Gluconic acid, Baicalein, FA 18:1+3O, 1,3-Dicaffeoylquinic acid, Saikosaponin a and 6-Gingerol, which mainly belong to carbohydrates, hydroxy acids, organic acid, carboxylic acids, flavonoids, phenylpropanoids, terpenoids and phenols.

**Table 3:** The main compounds in GEO.

Name	Formula	Class	Relative amount (%)
Cellobiose	C12H22O11	Carbohydrates and derivatives	33.12
D- (+)-Malic acid	C4H6O5	Hydroxy acids and derivatives	7.36
Mannose	C6H12O6	Carbohydrates and derivatives	3.68
Fumaric acid	C4H4O4	Organic acids and derivatives	3.42
Citric acid	C6H8O7	Carboxylic acids and derivatives	2.73
Glycitein	C16H12O5	Flavonoids	2.47
d-Gluconic acid	C6H12O7	Organic acids and derivatives	2.4
Baicalein	C15H10O5	Flavonoids	2.16
FA 18:1+3O	C18H34O5	Miscellaneous	2.07
1,3-Dicaffeoylquinic acid	C25H24O12	Phenylpropanoids	2.04
(2*S*,3*S*,4*S*,5*R*,6*R*)-6-[[(3*S*,4*R*,6a*R*,6b*S*,8a*S*,14b*R*)-4-(hydroxymethyl)-4,6a,6b,11,11,14b-hexamethyl-8a-[(2*S*,3*R*,4*S*,5*S*,6*R*)-3,4,5-trihydroxy-6-(hydroxymethyl)oxan-2-yl]oxycarbonyl-1,2,3,4a,5,6,7,8,9,10,12,12a,14,14a-tetradecahydropicen-3-yl]oxy]-3,4,5-trihydroxyoxane-2-carboxylic acid	C42H66O15	Terpenoids	2.03
Saikosaponin a	C42H68O13	Terpenoids	1.55
9-hydroxy-10,12-octadecadienoic acid	C18H32O3	Aliphatic acyl	1.51
6-Gingerol	C17H26O4	Phenols	1.39
4-hydroxybenzaldehyde	C7H6O2	Phenols	1.36
LPC 16:0	C24H50NO7P	Lipids	1.23
Inermin	C16H12O5	Flavonoids	1.14
Apigenin	C15H10O5	Flavonoids	1.11

### 3.2 Effects of GEO supplementation on serum cytokines and IgG of weaned piglets

**Fig. 2:**
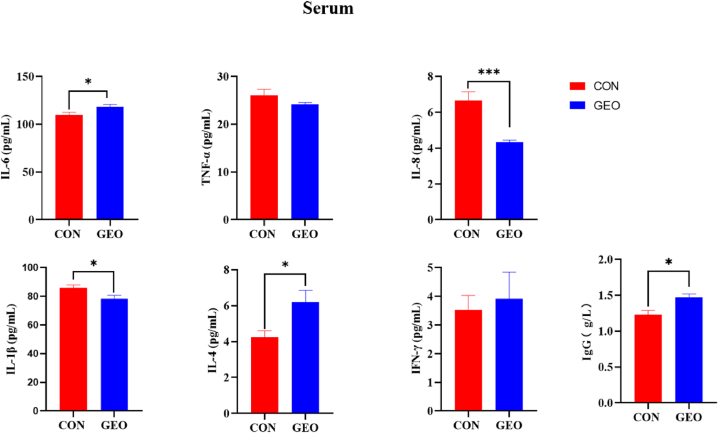
The effects of GEO supplementation on serum cytokine levels. Values are presented as means ± SEM, n = 6. *P < 0.05, ***P < 0.001, respectively. IL-6, Interleukin 6; IL-8, Interleukin 8; IL-1β, Interleukin 1 beta; IL-4, Interleukin 4; TNF-α, Tumor necrosis factor-alpha; IFN-γ, Interferon-gamma; IgG, Immunoglobulin G.

To reveal the effect of GEO supplementation on immune response, six common cytokine markers and IgG in serum were measured (Fig. 2). Compared with piglets fed basal diet, piglets supplemented with GEO had higher serum levels of IL-6, IL-4 and IgG (*P* < 0.05) but lower serum levels of IL-8 (*P* < 0.001) and IL-1β (*P* < 0.05).

### 3.3 Effects of GEO supplementation on immune-related genes in jejunum and ileum mucosa of weaned piglets

**Fig. 3:**
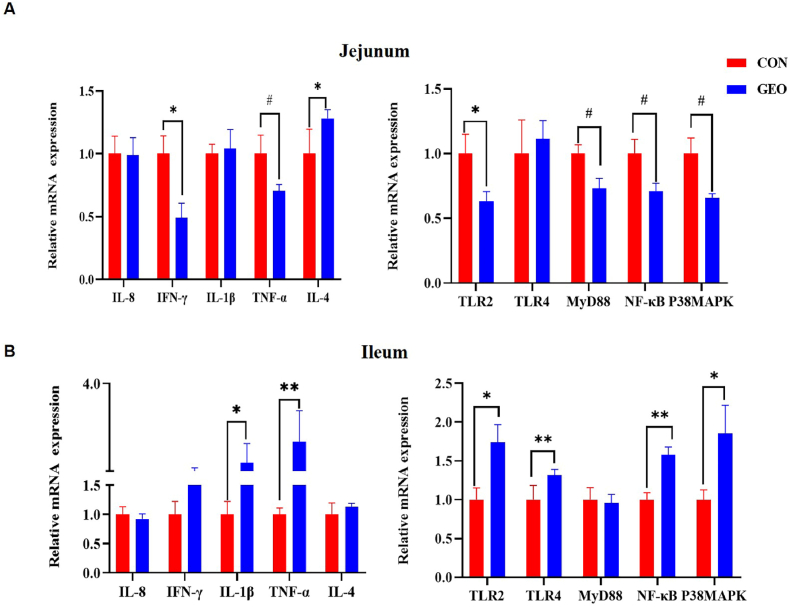
The effects of GEO supplementation on the mRNA expression of cytokines and immune pathway in jejunum **(a)** and ileum **(b)** of weaned piglets. Values are presented as means ± SEM, n = 6. #*P* < 0.1, *P < 0.05 and **P < 0.01, respectively. IL-8, Interleukin 8; IL-1β, Interleukin 1 beta; IL-4, Interleukin 4; TNF-α, Tumor necrosis factor-alpha; IFN-γ, Interferon-gamma; TLR, Toll-like receptor; MyD88, myeloid differentiation 88; NF-κB, Nuclear factor-kappa B; P38MAPK, Mitogen activated protein kinase.

To better understand the effect of GEO supplementation on immune response and inflammation, the mRNA expression of cytokines and immune pathway in the jejunum and ileum mucosa of weaned piglets were analyzed. Compared with the control group, GEO supplementation markedly down-regulated the mRNA expression of *IFN-γ, TLR2* (*P* < 0.05), and tended to down-regulated the mRNA expression of *TNF-α*, *MyD88*, *NF-κB*, and *P38 MAPK* (*P* < 0.10) in jejunal mucosa of weaned piglets. While dietary GEO significantly up-regulated the mRNA expression of *IL-4* in the jejunal mucosa of weaned piglets (*P* < 0.05) (Fig. 3A). In the ileal mucosa, the mRNA expression of *TNF-α* (*P* < 0.01), *TLR4*, *NF-κB* (*P* < 0.01), *P38 MAPK*, *TLR2* and *IL-1β* (*P* < 0.05) were significantly up-regulated in the GEO group than those in the control group (Fig. 3B).

### 3.4 Effects of GEO supplementation on composition of intestinal microbiota

**Fig. 4:**
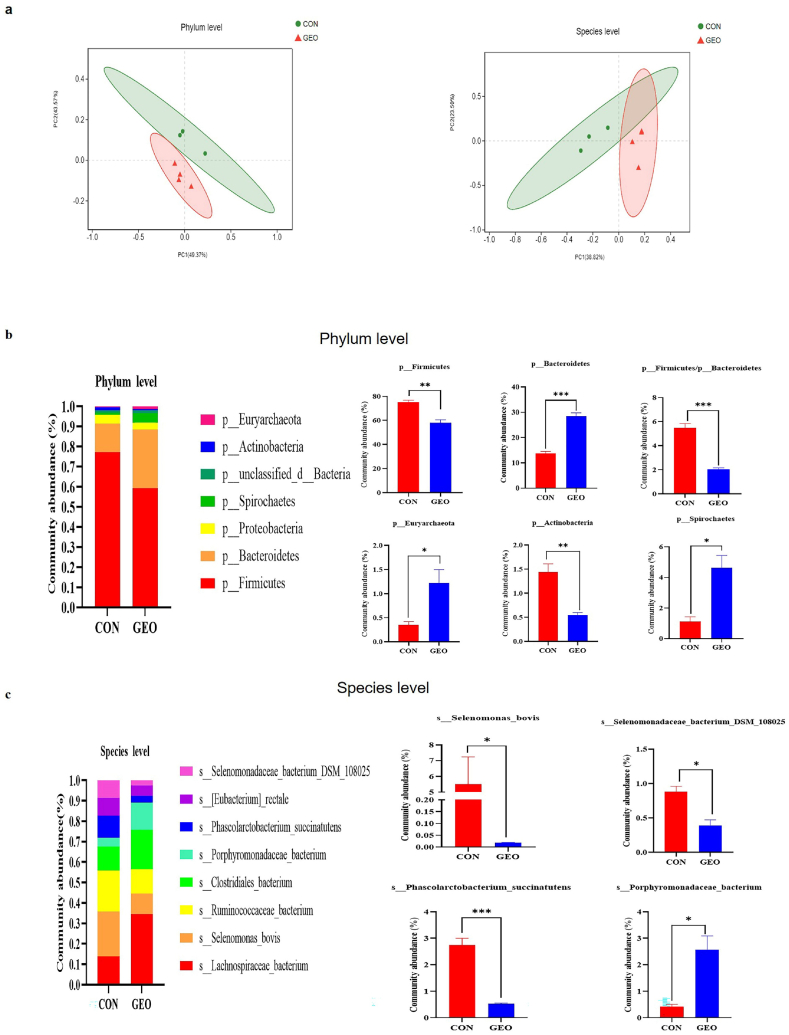
The effects of GEO supplementation on microbial community structure **(A)** and microbiota composition in the phylum **(b)** and species level **(c).** N = 3 in the control group, n = 4 in the GEO group, Data are presented as mean ± SEM. #*P* < 0.1, *P < 0.05, **P < 0.01, and ***P < 0.001, respectively.

To assess the overall structure of intestinal microbiota in response to GEO supplementation, principal coordinates analysis (PCoA) based on the Bray-Curtis distance was used to detect the structure of colonic content in weaned piglets (Fig. 4A). One sample in the control group was far away from the other three samples, consequently, it was deleted in the next analysis. The control group and GEO group were observed distinct bacterial structures at phylum level (*R* = 0.630, *P* = 0.021) and species level (*R* = 0.870, *P* = 0.021). In the phylum level, the majority of microbiota in all samples was composed of four predominant bacterial phyla: *Firmicutes*, *Bacteroidetes*, *Proteobacteria* and *Spirochaetes*. The result further demonstrated that GEO supplementation increased the relative abundance of *Bacteroidetes, Spirochaetes and Euryarchaeota* (*P* < 0.05). Conversely, the relative abundances of *Firmicutes* and *Actinobacteria* were decreased in the GEO group compared with that in the control group *(P < 0.05)*. Furthermore, the *Firmicute* to *Bacteroidetes* ratio (F/B) was highly significant decreased (*P* < 0.001) (Fig. 4B). At species level, the main microbiota in all samples were: *Lachnospiraceae_bacterium*, *Selenomonas_bovis*, *Ruminococcaceae_bacterium*, *Clostridiales_bacterium*, *Porphyromonadaceae_bacterium* and *Phascolarctobacterium_succinatutens*. After detailed analysis, the relative abundances of *Selenomonas_bovis*, *Selenomonadaceae_bacterium_DSM_108025* and *Phascolarctobacterium_succinatutens* (*P* < 0.05) in the GEO group were lower than that in the control group (Fig. 4C), while the abundance of *Porphyromonadaceae_bacterium* was higher in the GEO group compared with that in the control group.

### 3.5 Effects of GEO supplementation on virulence factor (VFDB) and correlations between the deferentially abundant taxa and predicted deferentially abundant pathways

**Fig. 5:**
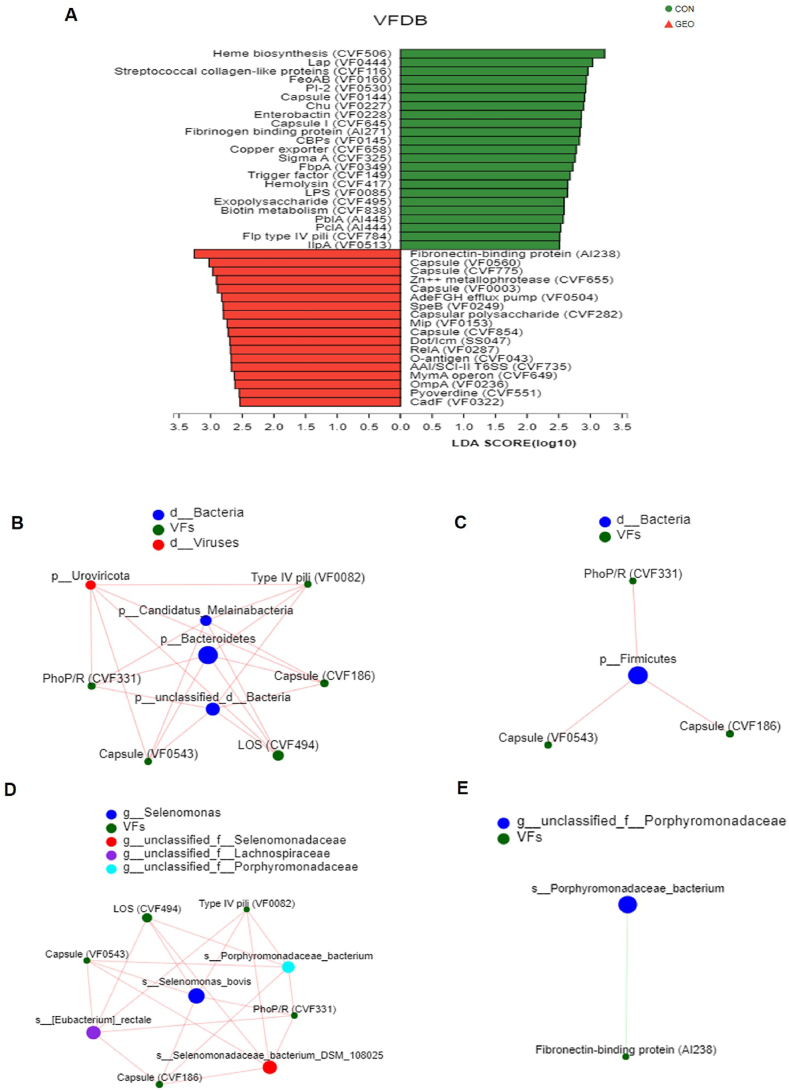
Effects of GEO supplementation on VFDB at virulence factor level (a) and correlations between the differentially abundant taxa and predicted differentially abundant pathways of the VFDB in the Virulence factors (b, c, d, and e). (b) CON at phylum level, (c) GEO at phylum level, (d) CON at species level, (e) GEO at species level n = 3 in the control group; n = 4 in the GEO group. All nodes are shown in the same color. The color of the line indicates a positive or negative correlation, with red indicating a positive correlation and green indicating a negative correlation. The thickness of the line indicates the magnitude of the correlation coefficient value, and the thicker the line, the higher the correlation. (For interpretation of the references to color in this figure legend, the reader is referred to the Web version of this article.)

To gain sight into whether the altered microbiota relative abundance would lead to the function alteration. Linear discriminant analysis (LDA) (Fig. 5A) was conducted to identify fecal microbial functional annotation in VFDB that accounted for the greatest differences between the two groups. The gene catalog was assigned to VFDB database based on the Virulence factors. Heme biosynthesis (CVF 506), Lap (VF 0444), Streptococcal collagen-like proteins (CVF 116), FeoAB (VF 0160), PI-2 (VF 0530) and Chu (VF 0227) were specific in the control group, while Fibronecin-binding protein (A1238), Zn^++^ metallophrotease (CVF665), AdeFGH effux pump (VF0504) are predominant in the GEO group.

To explore the correlations not only between species and species, but also between species and functional categories, Spearman's rank correlation analysis was done with the two factors correlation analysis. The control group and the GEO group were respectively analyzed. On the other hand, the correlations in the top 10 species and the VFDB function level (Virulence factors) were explored. In the Phylum level, *Bacteroidetes, unclassified_d_Bacteria*, *Candidatus_Melainabacteria* and *Uroviricota* were positively associated with LOS (CVF494), Capsule (CVF186) and Capsule (VF0543) in the control group (Fig. 5B). *Firmicutes* had positive correlations with Capsule (CVF186), Capsule (VF0543) and PhoP/R (CVF331) in the GEO group (Fig. 5C). At species level, *Uroviricota*, *Candidatus_Melainabacteria*, *p_BacteroidetesPhoP/R* (CVF331) and *unclassified_d_Bacteria* were positively linked with LOS (CVF494), Capsule (CVF186), Type IV pili (VF0082) and Capsule (VF0543) in the control group (Fig. 5D). Meanwhile, *Porphyromonadaceae_bacterium* had a negative correlation with Fibronectin-binding protein (AI238) in the GEO group (Fig. 5E). Above all, we found the correlations in the control group were more complex than that in the GEO group, and the GEO group lacked a functional pathway associated with Viruses.

### 3.6 Effects of GEO supplementation on the SCFAs contents, and the mRNA expression of GRP41 and GRP43

**Fig. 6:**
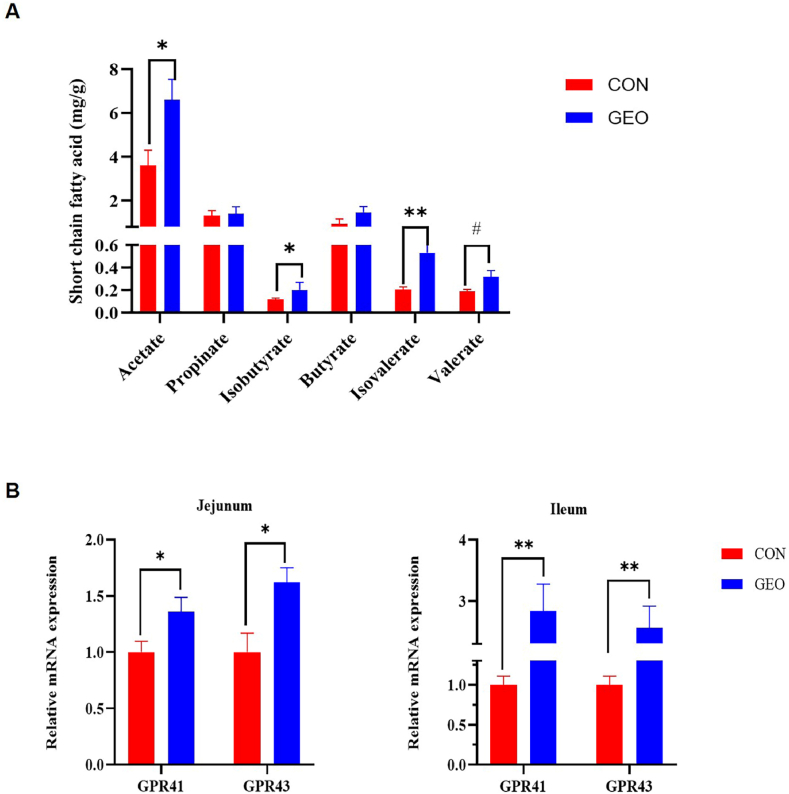
The effects of GEO supplementation on the SCFA concentration from colonic content (a), GPR41 and GPR43 in the jejunum and ileum (b) of weaned piglets. GPR, G-protein coupled receptor. Data are presented as mean ± SEM, n = 6. #*P* < 0.1, *P < 0.05 and **P < 0.01, respectively.

To detect the effect of GEO supplementation on SCFAs production, we measured the concentrations of SCFAs concentrations from colon contents (Fig. 6A). Compared with the control group, weaned piglets in the GEO group had significantly higher acetate, isobutyrate, isovalerate and valerate (*P* < 0.10) in the colon content. Since SCFAs are recognized by the G-protein coupled receptors, including GPR41 and GPR43 [24], we investigated the expression pattern of these receptors in jejunum and ileum mucosa by RT-qPCR. As shown in Fig. 6B, the mRNA expression of *GPR41* and *GPR43* were up-regulated in both jejunum and ileum mucosa in GEO group compared with that in the CON group (*P* < 0.05).

### 3.7 Correlation between microbiota, cytokine content and SCFA

**Fig. 7:**
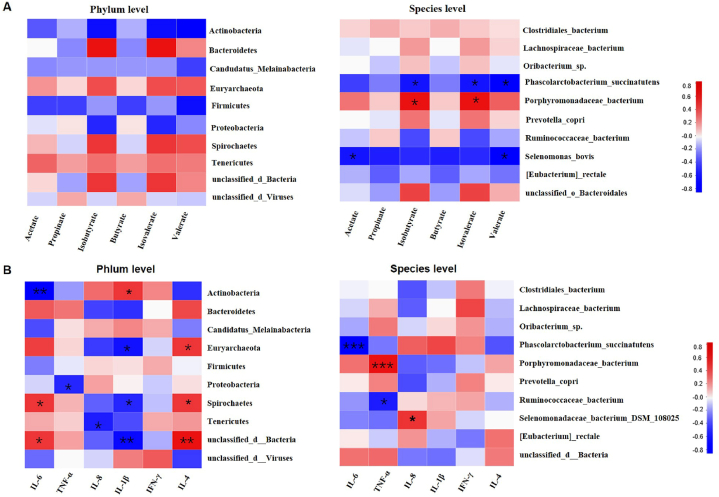
The heat map represents the correlation between colonic microbiota and colonic SCFAs, cytokines and IgG in GEO and control group weaned piglets. *P < 0.05, **P < 0.01, ***P < 0.0001.

To determine whether the changed in gut microbiota had an association with SCFAs, the Spearman correlation analyses were carried out. Remarkably, at species level, *Porphyromonadaceae_bacterium* was positively associated with isobutyrate and isovalerate, however, *Selenomona_bovis* had a negative association with acetate and propinate. In addition, *Phascolarctobacterium_succinatutens* was negative association with isobutyrate, isovaterate and valerate (Fig. 7A).

To gain sight into whether the altered bacteria genus had link with serum cytokine contents, Spearman correlation analysis between bacteria taxa and cytokine contents was employed. In the phylum level, the *Spirochaetes* had a positive association with IL-6 and IL-4, but had a negative with IL-1β. *Acinobacteria* was positively correlated with IL-1β, while negative with the IL-6; *Proteobacteria* had negative association with TNF-α. In addition, *Euryarchaeota* had a positive correlation with IL-4, but a negative correlation with IL-1β; *Tenericutes* was negative with IL-8; And *unclassed_d_bacteria* were positive with IL-4, but negative with IL-1β. At species level, *Phascolarcolarctobacterium_succinatutens* and was negative with IL-6; *Ruminococcaceae_bacterium* was negative with TNF-α, respectively. While, *Porphyromonadaceae_bacterium* had a positive correlation with TNF-α and *Selenomonadaceae_bacterium_DSM_*108025 had a positive correlation with IL-8 (Fig. 7B).

## 4 Discussion

To identify the main bioactive compound present in GEO in this study, the UHPLC**-**QE**-**MS was operated, which is one of the best tools to identify and quantify the constituents of plant products because of its simplicity, rapidity, accuracy, and efficiency [25]. The result shows that the main chemical classes were carbohydrates, hydroxy acids, organic acid, carboxylic acids, flavonoids, phenylpropanoids, terpenoids and phenols. This result corroborates with Dhifi who found except the terpenes, essential oils also contain various chemical classes including alcohols, ethers, aldehydes, ketones, esters, amines, amides, and phenols [26]. While, our results are different with Mugao LG who reported that the organosulfur, aromatic hydrocarbons, carbonates and carbaldehyde are the main compounds in the GEO [27]. The difference maybe because of the different GEO production process. Mugao LG stored the GEO in sealed vials at 4 °C awaiting the GC-MS analysis, while the GEO in our study first attached to porous multigrain starch balls made by enzyme and then to be tested by the UHPLC**-**QE**-**MS analysis. And the GEO in our study has undergone a deodorization process which is probably why there are no thioethers in the constituent.

A well-developed immune system is essential for protecting the host from pathogenic microorganisms. Cytokine content is an excellent tool for motoring immune response linked with inflammation and immunity. Interestingly, in this study, compared with piglets in control group, a significant increase in serum levels of IL-6, IL-4, IgG and a profoundly reduction in serum levels of IL-1β and IL-8 were observed in piglets fed the basal diet supplemented with GEO. The present results are similar to the former studies that allicin could reduce the levels of TNF-α and IL-1β in serum, IL-1β in colon and increase the levels of IL-4 in serum or allicin significantly inhibited spontaneous and TNF-α-induced secretion of IL-1β, IL-8, IP-10 and MIG, and inhibited mRNA expression of IL-8 and IL-1β in two different cell lines in a dose-dependent manner [28,29]. The results indicated that the product of GEO may promote the immune response of animals.

NF-κB and P38MAPK are not only inflammatory pathways, but also could activate immune responses [30]. The TLR4/MyD88 signaling pathway is the upstream gatekeeper of NF-κB [31]. TLR4 is a family of pathogen recognition receptors (PRRs) that orchestrate the host immune system through MyD88 to induce pro-inflammatory cytokines via NF-κB [32]. The effect of GEO on cytokine mRNA expressions in the jejunum mucosa was firstly study. The current results showed that GEO supplementation down-regulated the mRNA expression of *IFN-γ* and *TLR2*, *TNF-α*, *MyD88*, *NF-κB*, and *P38MAPK*, while significantly up-regulated the mRNA expression of *IL-4* in the jejunal mucosa. It is reported that allicin, a product of garlic, could inhibit neuroinflammation by suppressing the reactive oxygen species (ROS) production and inhibition of TLR4/MyD88/NF-κB, P38 and JNK pathways [33], which demonstrated that the product of garlic own the role of anti-inflammation [34]. Consequently, it was supposed that GEO may promote immunity at least partly by inhibiting the inflammation occurrence in the jejunum mucosa, which may account for the higher concentration of IL-4 and the decreased concentration of IL-8 in the serum of weaned piglets in the GEO group compared with that in the control group in this study.

Previous researches reported that time-related damage of epithelial function occurred faster in the jejunum than in the ileum [35] and in a pig small transplantation experiment, which used cold ischemic and periods ranging from 0 to 12 h, estimated (based on the light microscopy) that the ileum is more resistant to preservation injury than jejunum [36]. Ulcerative colitis (UC) and Crohn's disease (CD) causes inflammation mostly in the ileum, colon, or both [37]. Consequently, it was supposed the stress always occurred in the ileum and colon. These may indicate that the ileum provokes an immune response more easily than jejunum. Therefore, in the ileum mucosa, dietary GEO up-regulated the mRNA expression of *TNF-α*, *TLR4*, *NF-κB*, *P38 MAPK*, *TLR2* and *IL-1β*. Consequently, it was supposed that the ileum of weaned piglets suffered weaning stress in the present study, therefore dietary GEO might activate the TLR4/MyD88/NF-κB signaling pathway in the ileum mucosa, thereby induced an immune response to upregulate the mRNA expression of *IL-1β* and *TNF-α* to stimulate the immune cells to secret immune IgG. Correspondingly, the serum IgG and IL-6 concentration of weaned piglets was higher in the GEO group, which indicated that dietary GEO might promote the immune response of weaned piglet in the ileum mucosa of weaned piglets.

Intestinal microorganisms have widely biological effects on the growth and health of humans and animals [38,39], the interaction between members of the intestinal microflora and the interaction between intestinal microorganisms and hosts can regulate the biological processes that are vital to the health of the host. In this study, a distinct bacterial structure at phylum level and species level were observed between the control group and GEO group, indicating that GEO supplementation may alter the microbial community composition in colon of weaned piglets. It was contradictory with the previous studies, which showed there were no bacterial structures alteration on the fish [40] and mice [41] after dietary garlic oil or garlic extract, these distinctive results may because of the different experimental animals and thus the different physiological responses to GEO.

Next, how the microbe changed at phylum and species level were explored. In the phylum level, it showed that GEO supplementation increased the relative abundance of *Bacteroidetes* and *Euryarchaeota*, conversely, the relative abundances of *Firmicutes* and *Actinobacteria* were decreased compared with that in the control group. Furthermore, the *Firmicute* to *Bacteroidetes* ratio (F/B) was decreased. It has been reported that *Bacteroides* are also involved in immune system development [42,43] and maintenance of gut microbiota balance [44,45]. An increase in Firmicutes/Bacteroidetes ratio typically associated with proinflammatory condition [45], which may demonstrate that the decreased Firmicutes/Bacteroidetes ratio may reduce the inflammation occurrence. And the relative abundance of *Spirochaetota* in this study was increased*.* A study showed that *Spirochaetota* in fecal microbial community of Tibetan piglets in yellow dysenteric and diarrhea group was significantly decreased compared with that in the control group, which may indicate that *Spirochaetes* may reduce the diarrhea situation of piglets [46]. Dietary GEO decreased the relative abundance of *Actinobacteria.* Patients with inflammatory bowel disease [47] have high levels of *Actinobacteria* at the colon, which may indicate *Actinobacteria* was thought to be linked with inflammation. At species level, the abundance of *Porphyromonadaceae_bacterium* was higher in the GEO group compared with that in the control group, *Porphyromonadaceae_bacterium*, a species within the phylum *Bacteroidetes,* which was reported *Porphyromonadaceae*, *Rikeneliaceae*, and *Lachnospiraceae* significantly decreased in the UC patient in the family level [48]. Consequently, it was speculated *Porphyromonadaceae_bacterium* had anti-inflammatory effect. Taken together, the results indicated that dietary GEO increased the relative abundance of anti-inflammatory bacteria composition, while decreased the relative abundance of harmful bacterium composition. These results are consistent with Filocamo who reported garlic may contribute to the growth of these beneficial bacterial species in the gut [49]. Therefore, GEO promote immune response and reduce inflammation occurrence partly due to the change in colon microbiota.

To explore how the microbial community affect the immune function, LDA analysis was operated to identify colon microbial functional annotation VFDB that accounted for the greatest differences between the groups. The gene catalog was assigned to VFDB database based on the Virulence factors to detect the correlation between the microbiota with immunity. So far, it was found Heme biosynthesis (CVF 506), Lap (VF 0444), Streptococcal collagen-like proteins (CVF 116), FeoAB (VF 0160), PI-2 (VF 0530) and Chu (VF 0227) were specific in the control group. Bacterial pathogens have adopted considerable mechanisms for acquiring iron from host proteins during an infection [50], which may not only decrease the immune defenses of the host but also promote the virulence of pathogenic bacteria. The streptococcal collagen-like proteins are widely present in pathogenic streptococci [51]. Pl-2 may trigger an inflammatory [52]. Fibronecin-binding protein (A1238), Zn++ metallophrotease (CVF665), Capsular polysaccharides (CVF 282) are predominant in the GEO group. Fibronectin plays a vital role in regulating immune cell migration and immune cell adhesion. Importantly, fibronectin also serves as a common target for bacterial adhesins in the gut [53]. Fibronecin-binding protein (FBP) could be regarded as a potential candidate for the therapeutic intervention or as nutraceutical for the prophylaxis of enteric infections [54]. Taken together, the VFDB functional pathways in the control group are mainly associated with the inflammation and pathogenicity, while in the GEO group are principally linked with the immune and protect the host from the diseases. These results are similar to previous studies that demonstrate garlic or its active ingredients may inhibit the harmful virulence factor [[55], [56], [57]].

To reveal the correlations not only between species and species, but also between species and functional categories, Spearman's rank correlation analysis was operated with the two factors correlation analysis. In this study, the association between gut microbiota and cytokine production was observed. Some of the bacteria genera such as *Actinobacteria*, *Spirochaetes* had positive associations with cytokines production, suggesting that the immunomodulatory effects of GEO may be at least partially through modulating of intestinal microbiota.

Intestinal flora ferments dietary fiber to produce organic acid, gas and other metabolites and a large number of short-chain fatty acids. The SCFA are produced primarily in the colon where the biomass is the highest [[58], [59], [60]]. And it has been well established that SCFAs activate G-protein coupled receptors (GPR41 and GPR43), to regulate immune functions and control intestinal inflammation [24]. In the current study, compared to the piglets fed with basal diet, weaned piglets fed a diet with GEO had significantly higher acetate, isovalerate and valerate in the colon content. The mRNA expression of GPR41 and GRP43 were up-regulated in both jejunum and ileum mucosa in the present study. This data is in line with Leong et al. who found Patchouli Essential Oil could stimulate the SCFAs production and the key SCFA-sensing receptors (GPR41 and GPR43) [61], consequently, it was supposed that GEO decreased the inflammation occurrence promoted the immunity partly because of the increased SCFAs concentrations, which may partly explain the anti-inflammation effect in jejunum and the immune response in ileum.

Next, to determine whether the changed gut microbiota had an association with SCFAs and cytokines, the Spearman correlation analyses were carried out at phylum and species level. Spearman correlation analysis indicated complex correlations between altered microbiota vs serum cytokine levels and SCFAs production. Generally, we observed an association between microbiota and cytokine production, which further confirmed that microbes could affect immunity.

## 5 Conclusion

Generally, metagenomic analysis and SCFA results demonstrated that GEO supplementation altered the structure of intestinal microbiota, their metabolites and the VFDB functional pathway. These data suggested that immune regulation effects of GEO may be associated with the shift in microbiota composition, their metabolites and VFDB functional pathway in response to these diets. Together, our results suggested that immune regulation effects of GEO may be associated with the microbiome compositions in response to GEO.

## Funding

This paper was supported by the Key Collaborative Research Program of the Alliance of International Science Organizations (Grant No. ANSO-CR-KP-2021-10).

## Author contribution statement

Bei Cheng: Performed the experiments; Analyzed and interpreted the data; Contributed reagents, materials, analysis tools or data; Wrote the paper. Mingyong Huang: Performed the experiments. Tiantian Zhou: Qingqing Deng: Analyzed and interpreted the data. Teketay Wassie: Contributed reagents, materials, analysis tools or data. Tao Wu: Xin Wu: Conceived and designed the experiments.

## Data availability statement

Data associated with this study has been deposited at China National Microbiology Data Center (NMDC) under the accession number (https://nmdc.cn/resource/genomics/sample/detail/NMDC10018244), https://doi.org/10.6084/m9.figshare.21929793.v1.

## Declaration of competing interest

The authors declare that they have no known competing financial interests or personal relationships that could have appeared to influence the work reported in this paper.
